# From Beauty to Botulism: A Case Report Highlighting the Rare Risk of Botox Administration

**DOI:** 10.7759/cureus.54090

**Published:** 2024-02-12

**Authors:** Jordan Richardson, Shannon Viviano

**Affiliations:** 1 Emergency Medicine, Corewell Health William Beaumont University Hospital, Royal Oak, USA; 2 Emergency Medicine, Corewell Health Beaumont Troy Hospital, Troy, USA

**Keywords:** case report, antitoxin, botox, botulism, iatrogenic

## Abstract

Botox (onabotulinumtoxinA) is a pharmaceutical approved by the Food and Drug Administration (FDA) for use in both cosmetic and therapeutic applications. Despite its increasing use worldwide, Botox carries a rare but potentially life-threatening risk of iatrogenic botulism. This condition, although treatable with antitoxin if promptly recognized, presents a diagnostic challenge to healthcare providers due to its rarity, lack of awareness, and diverse clinical presentations. Here, we present a case of iatrogenic botulism from Botox injections administered in Istanbul, Turkey, in a healthy 47-year-old female. Prompt administration of antitoxin led to remarkable clinical improvement. This case underscores the importance of vigilance among healthcare providers in recognizing and promptly treating iatrogenic botulism, particularly in patients with recent Botox use. Given that the majority of reported cases of iatrogenic botulism occur outside the United States, this case raises concerns about the need for stricter regulations and oversight of Botox administration worldwide.

## Introduction

In the 1940s, botulinum toxin was considered one of the deadliest substances known to humanity, even being considered for potential weaponization during World War II [1]. Derived from the spore-forming bacterium Clostridium botulinum, this potent neurotoxin disrupts the release of acetylcholine from peripheral nerve cells, leading to skeletal muscle paralysis and, ultimately, respiratory failure and death. Botulism, categorized into foodborne, wound, and infantile forms, poses a significant threat to human health [2]. Interestingly, pharmaceutical advancements have led to the iatrogenic emergence of botulism, notably through the use of Botox (onabotulinumtoxinA). Originally discovered for its therapeutic potential in treating strabismus by an ophthalmologist in the 1970s, Botox has since gained approval from the United States Food and Drug Administration (FDA) for various clinical and cosmetic purposes including urinary incontinence due to detrusor overactivity, migraines, cervical dystonia, and axillary hyperhidrosis [3,4]. Despite its widespread use and generally favorable safety profile, systemic circulation of the toxin can precipitate iatrogenic botulism, mirroring other forms of botulism toxicity with manifestations such as cranial nerve palsies, flaccid paralysis, and respiratory depression [4]. The popularity of Botox has surged in recent years, with approximately 7.4 million individuals in the United States alone receiving the treatment in 2022 [5]. 

Documenting iatrogenic botulism cases is crucial for raising awareness and enabling prompt diagnosis and treatment. Here, we present a case of iatrogenic botulism in a 47-year-old female following Botox injections, highlighting the clinical features, diagnostic challenges, and treatment associated with this uncommon condition.

## Case presentation

A 47-year-old female with no significant medical history presented to the emergency department with symptoms of blurred vision, difficulty swallowing, hoarse voice, shortness of breath, and weakness. Approximately three weeks earlier, she had received Botox injections in her forehead and bilateral axilla while in Istanbul, Turkey. The forehead treatment involved 50 units of Dysport (abobotulinumtoxinA), while the axillary treatment utilized a total of 90 units of Dysport. Her symptoms initially manifested as blurred vision and diplopia, progressing to include dysphagia and dysphonia. Despite seeking care at an emergency department in Istanbul, she was discharged without a diagnosis. Upon returning to the United States, she presented to our facility with symmetric, progressive descending weakness and reported shortness of breath, although she was not in respiratory distress and remained hemodynamically stable.

A detailed neurological examination revealed an alert and oriented patient without dysarthria, with a hoarse voice. Visual acuity was normal, with no observed visual field deficits or ophthalmoplegia, although mild bilateral ptosis was noted. Facial sensation was normal to light touch and pinprick, with minimal movement in the right frontalis muscle and absent movement on the left side. The patient's hearing was grossly intact, with normal soft palate and tongue movement and a normal gag reflex. Muscle strength in the trapezius and sternocleidomastoid muscles was normal, except for bilateral iliopsoas weakness. The deep tendon reflexes in both upper and lower extremities were normal and symmetrical bilaterally, and the Babinski tests yielded negative results on both sides. Sensation to pinprick, light touch, and vibration in both hands and feet was normal, as were finger-to-nose, heel-to-shin, fine hand movement, and rapid alternating movement tests.

Routine laboratory examinations, including a complete blood count and basic metabolic panel, revealed results within normal ranges, as illustrated in Table 1.

**Table 1:** Basic laboratory results obtained in the emergency department. RDW-CV, red cell distribution width; MCH, mean corpuscular hemoglobin; MCHC, mean corpuscular hemoglobin concentration; MCV, mean corpuscular volume; eGFR, estimated glomerular filtration rate

Complete blood count	Results	Reference
WBC	7.3	3.3-10.7 bil/L
RBC	4.43	3.87-5.08 tri/L
Hemoglobin	13.7	12.1-15.0 g/dL
Hematocrit	41.8	35.4%-44.2%
MCV	94	80-100 fL
MCH	31	28-33 pg
MCHC	33	32-35 g/dL
RDW-CV	13	12%-15%
Platelets	326	150-400 bil/L
Nucleated red blood cells	0.0	<=0.0%
Neutrophils	4.2	1.6-7.2 bil/L
Lymphocytes	2.2	1.1-4.0 bil/L
Monocytes	0.6	0.0-0.8 bil/L
Eosinophils	0.1	0.0-0.5 bil/L
Basophils	0.1	0.0-0.1 bil/L
Immature granulocytes	0.05	0.00-0.03 bil/L
Immature granulocyte %	0.7	0%-0.6%
Basic metabolic panel		
Sodium	141	135-145 mmol/L
Potassium	3.8	3.5-5.2 mmol/L
Chloride	107	98-111 mmol/L
Carbon dioxide (CO_2_)	25	20-29 mmol/L
Anion gap	9	5-17
Glucose	87	60-99 mg/dL
Blood urea nitrogen (BUN)	14	7-25 mg/dL
Creatinine	0.66	0.50-1.10 mg/dL
eGFR by creatinine	109	mL/min/1.73 m^2^
Calcium	9.3	8.5-10.5 mg/dL

**Figure 1:**
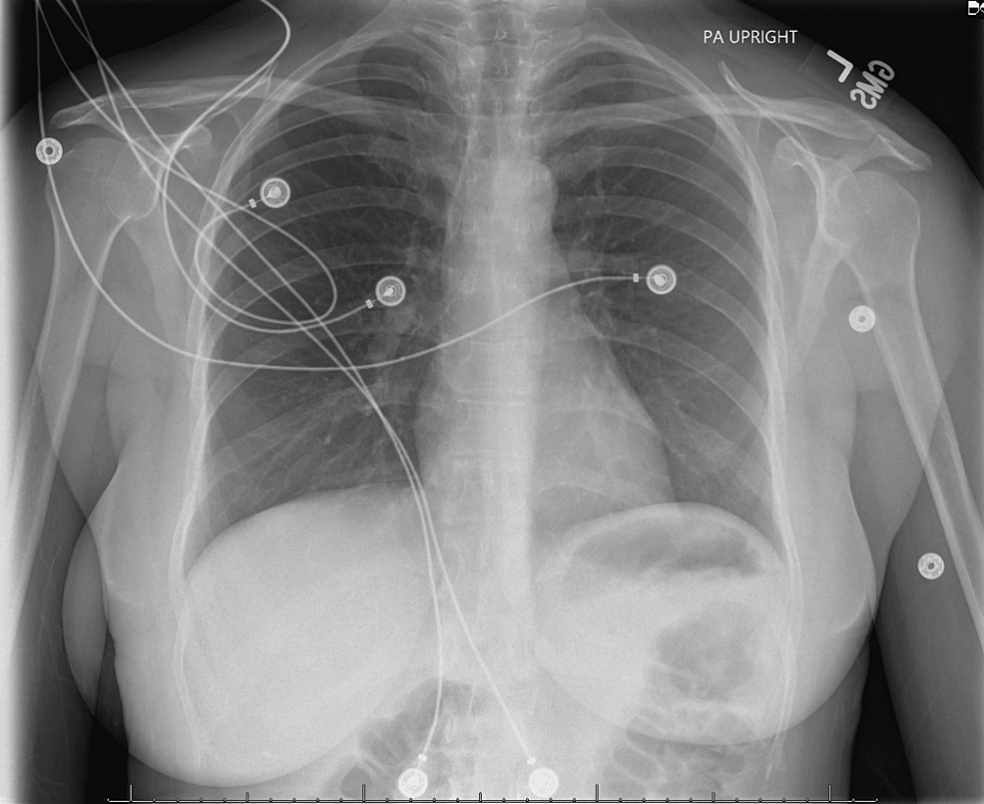
A single-view chest radiograph that was obtained in the emergency department.

Testing for myasthenia gravis antibodies was negative, and the findings from the chest X-ray appeared normal (Figure 1).

A clinical diagnosis of iatrogenic botulism was established. Our team discussed the case with the Centers for Disease Control (CDC), and the antitoxin was promptly airlifted from Atlanta and delivered to our facility within hours. Meanwhile, the patient was admitted to the intensive care unit (ICU) for close monitoring until the antitoxin arrived. Her lower extremity weakness worsened during her ICU stay, making ambulation difficult. Her condition deteriorated before the administration of antitoxin.

Twelve hours after her initial presentation to the hospital, the patient received 198 mL of Botulism Antitoxin Heptavalent (A, B, C, D, E, F, G) in 0.9% normal saline, along with 20 mL of botulism immune globulin (Human) 100 mg IV SOLR 1,000 mg. She did not experience any reported adverse reactions. Clinical improvement was observed almost immediately after the administration of antitoxin. She was observed in the ICU for one day and discharged on hospital day 2. The patient was followed up with one month after discharge and remains symptom-free.

## Discussion

Diagnosing iatrogenic botulism is exceptionally challenging for healthcare providers. This difficulty stems from the disease's rarity, limited awareness among medical professionals, and the absence of readily available confirmatory tests. Recent literature reviews from 2017 reveal that botulism was frequently misdiagnosed as myasthenia gravis or Guillain-Barre syndrome [6,7]. This difficulty stems from the resemblance in signs and symptoms shared by these conditions and botulism, coupled with their increased prevalence. Additionally, there are no immediate laboratory tests to confirm the diagnosis. Serum and stool studies can be obtained, but often take several days to yield results [8]. Thus, iatrogenic botulism is a clinical diagnosis and the healthcare provider must have a high index of suspicion. To help clinicians, the CDC has outlined clinical criteria to help make the diagnosis, emphasizing specific signs and symptoms of cranial neuropathy, combined with the absence of fever [7]. A study in 2017 reported that these three clinical criteria were met in nearly 90 percent of patients with confirmed botulism [7]. We acknowledge the significant challenge in diagnosing this disease. Our case aims to inform healthcare providers about this lesser-known cause of botulism and to emphasize the importance of inquiring about recent Botox procedures in patients presenting with cranial nerve palsies and descending paralysis. 

The botulism antitoxin, designed for non-infant botulism, consists of equine serum antibodies to botulinum toxins A-G and is the only specific treatment for botulism [8]. Research from 2017 suggests that early antitoxin administration, within the initial 48 to 96 hours of symptom onset, correlates with lower mortality rates than delayed administration [9,10]. Despite our patient receiving antitoxin therapy eight days after symptom onset, her clinical condition rapidly improved post-treatment. In a 2016 case report by Fan et al., similar outcomes were observed, with two patients recovering swiftly from iatrogenic botulism after receiving antitoxin seven and nine days after the onset of symptoms [11].

The CDC maintains a supply of antitoxin, and healthcare professionals who suspect botulism in a patient should contact the 24-hour CDC botulism consultation service or call their local or state health department's emergency contact number to arrange antitoxin shipment.

While Botox injections can treat several medical conditions, controversies persist regarding its safety and regulatory oversight. In the United States, regulation of Botox administration by the FDA aims to ensure safety; however, cases of iatrogenic botulism persist globally, particularly in countries with less stringent regulations [11-15]. Our patient received Botox in Istanbul, Turkey, where previous cases of iatrogenic botulism have been reported [12,13]. This case highlights the importance of revisiting regulations surrounding Botox administration in countries where oversight may be less strict. 

Although iatrogenic botulism remains rare, increased global Botox utilization may lead to a rise in cases. Our experience underscores the importance of maintaining a high index of suspicion and obtaining thorough patient histories, particularly for those receiving treatment abroad. This vigilance is crucial for timely diagnosis and favorable patient outcomes. Lastly, to our knowledge, there are no epidemiological studies of iatrogenic botulism. This would be useful to provide insight into risk factors and clinical manifestations of iatrogenic botulism cases worldwide. 

## Conclusions

In conclusion, Botox injections continue to gain popularity each year, offering patients therapeutic and cosmetic benefits. However, it is imperative to acknowledge the potential risks associated with Botox administration, particularly the rare but serious complication of iatrogenic botulism. As evidenced by our case, prompt recognition and treatment are essential for favorable patient outcomes. Healthcare providers, particularly emergency physicians, should maintain a high index of suspicion for iatrogenic botulism in patients presenting with symptoms suggestive of cranial nerve palsies and descending muscle weakness, especially when there is a history of recent Botox use. Obtaining a comprehensive patient history, including travel details, is crucial for accurate diagnosis and timely intervention. Given that the majority of reported cases of iatrogenic botulism occur outside the United States, our findings emphasize the need for enhanced regulatory oversight and standardized training protocols for Botox administration globally. By implementing stricter regulations and promoting awareness among healthcare professionals, we can mitigate the risks associated with Botox administration and ensure the safety of patients worldwide.
